# Geocoding School and Student’s Home Addresses: Zandbergen Responds

**DOI:** 10.1289/ehp.11542R

**Published:** 2008-08

**Authors:** Paul A Zandbergen

**Affiliations:** Department of Geography, University of New Mexico, Albuquerque, New Mexico, E-mail: zandberg@unm.edu

Our research (Zandbergen and Green 2007) strongly suggests that the positional error in street geocoding is not random in direction and that the displacement along the street segment often occurs toward one side of the street because of incorrect address ranges in the street reference data. This “squeeze” effect is a common observation in geocoding using many different street data sets. The extent to which this occurs will vary among locales due to the varying quality of street reference data. The extent to which this introduces any bias into exposure assessments will vary with the specific pollution source being considered. Proximity to major roads with high traffic counts represents a particular case that is very much influenced by this effect, because many residential streets are perpendicular to major roads and address ranges often start at major roads. For other exposure scenarios, such as air pollution from industrial facilities, the “squeeze” effect will contribute to the overall positional error in geocoding and therefore to any misclassifications, but much less likely to any bias.

Whitsel et al. (2006) determined positional accuracy of geocoding by four commercial vendors through an empirical comparison of criterion locations and vendor-assigned coordinates. In the analysis of the effects of positional error on exposure classification, however, Whitsel et al. (2006) displaced address locations at random over a uniform distribution of the angle of displacement. This assumes there is no direction in the positional error and ignores the “squeeze” effect. Our studies show that the displacement of a street-geocoded location relative to the actual location of the residence is frequently along the street segment, and definitely not random in direction. For a large sample, the distribution of the direction of positional error may appear to be uniform because the directions of street segments often approximate a uniform distribution, unless the street segments follow a very strong grid pattern (e.g., Zimmerman et al. 2007). I therefore argue that the error propagation modeling used by Whitsel et al. (2006) substantially underestimates the effects of positional errors in geocoding on exposure classification for the particular scenario where exposure potential is determined on the basis of distance to major roads. Given the relatively complex nature of the spatial pattern in geocoding errors, we feel that determining misclassification based on actual geocoded locations is more reliable than employing simulated displacements.

I agree, however, that care should be taken in generalizing the results from our studies, and we do not think the 250–500-m range is the lower limit of spatial epidemiologic analysis in general. However, I challenge the commonly held assumption that positional errors in geocoding are relatively small, random in terms of their direction, and without positional bias.

Contrary to other forms of digital spatial data (e.g., land use, roads, census boundaries), geocoding results do not have an implicit scale, and hence the spatial resolution is not known without testing. Certainly, the scale of geocoded locations is not the same as the scale of the street reference data employed. The studies by Whitsel et al. (2006) and my own research represent the few attempts at determining the effective resolution of geocoding; that is, how reliable is spatial analysis of geocoding results at small distances? This effective resolution will depend on several factors, not the least of which is the variation across urban–rural gradients. For Orange County, Florida (Zandbergen 2007), I found that street geocoding of residential addresses using local street centerlines (1:5,000) resulted in a 90th percentile of the error distribution of 100 m. This corresponds very closely to the results of Cayo and Talbot (2003), who found a value of 96 m for urban areas and much larger values for suburban and rural areas. Based on this 90th percentile, typical street geocoding of residential addresses does not meet the positional accuracy standards for a 1:100,000 scale map based on the National Map Accuracy Standards (U.S. Bureau of the Budget 1947).

Higher-quality street reference data is expected to improve the positional accuracy of geocoding results, primarily through improved address ranges. However, I argue that the linear interpolation algorithm used in street geocoding presents inherent limitations, resulting in data that are insufficient for many large-scale applications. Higher-accuracy alternatives will need to be considered, including address points. In the address-point data model, residences and other buildings are represented as single points, with a much greater positional accuracy than is achievable using street geocoding. For a review and comparison of methods, see Zandbergen (2008). Several other jurisdictions, including Australia, Canada, and the United Kingdom, have already developed national address-point databases. In the United States, address-point databases are currently limited to selected areas, but this is expected to change. Epidemiologic researchers that employ geocoding would greatly benefit from being aware of alternatives to traditional street geocoding, in particular when analysis at fine spatial scales is required.
